# Supplementary material for: Haptic shared control improves neural efficiency during myoelectric prosthesis use
Source: Sci Rep. 2023 Jan 10;13:484. doi: 10.1038/s41598-022-26673-2 (PMC9832035; doi:10.1038/s41598-022-26673-2)
Supplement: Supplementary file 1 — Supplementary Information. [file 41598_2022_26673_MOESM1_ESM.docx]

Scientific Reports Supplementary Material

This section contains supplementary results from additional fNIRS optodes, which cover the left lateral, left medial, and right medial prefrontal cortex areas.

Left Lateral Prefrontal Cortex

The average total hemoglobin concentration in the left lateral prefrontal cortex was significantly greater than zero in the Standard condition (β=0.63, SE=0.30, p=0.04). The Vibrotactile condition was not significantly different from the Standard condition (β=-0.13, SE=0.35, p=0.71). Similarly, the Haptic Shared Control condition was not significantly different from the Standard condition (β=-0.51, SE=0.35, p=0.15). Experience with the task reduced cognitive load (significant change in total hemoglobin concentration: β=-0.08, SE=0.03, p=0.007). See Fig. S1 for a visualization of these results.

Supplementary Figure S1. The change in average total hemoglobin concentration in the left lateral PFC for each trial, where the individual points are the average for each trial in each condition, and the solid lines indicate the model's prediction.

The neural efficiency in the left lateral prefrontal cortex was significantly less than zero in the Standard condition (β=-0.97, SE=0.30, p=0.003). The Vibrotactile condition was not significantly different from the Standard condition (β=0.41, SE=0.36, p=0.27). However, the Haptic Shared Control condition significantly improved neural efficiency compared to the Standard condition (β=0.87, SE=0.36, p=0.02). Experience with the task improved neural efficiency: β=0.14, SE=0.03, p<0.001). See Fig. S2 for a visualization of these results.

Supplementary Figure S2. Neural efficiency in the left lateral PFC for each trial, where the individual points are the average for each trial in each condition, and the solid lines indicate the model's prediction

Left Medial Prefrontal Cortex

The average total hemoglobin concentration in the left medial prefrontal cortex not different from zero in the Standard condition (β=0.59, SE=0.39, p=0.14). The Vibrotactile condition was not significantly different from the Standard condition (β=-0.27, SE=0.51, p=0.58). Similarly, the Haptic Shared Control condition was not significantly different from the Standard condition (β=-0.48, SE=0.51, p=0.36). Experience with the task did not affect cognitive load (no significant change in total hemoglobin concentration: β=-0.03, SE=0.05, p=0.502). See Fig. S3 for a visualization of these results.

Supplementary Figure S3. The change in average total hemoglobin concentration in the left medial PFC for each trial, where the individual points are the average for each trial in each condition, and the solid lines indicate the model's prediction.

The neural efficiency in the left medial prefrontal cortex was significantly less than zero in the Standard condition (β=-0.69, SE=0.28, p=0.02). The Vibrotactile condition was not significantly different from the Standard condition (β=0.23, SE=0.36, p=0.52). Similarly, the Haptic Shared Control condition was not significantly different from the Standard condition (β=0.63, SE=0.36, p=0.09). Experience with the task improved neural efficiency: β=0.10, SE=0.03, p=0.001). See Fig. S4 for a visualization of these results.

Supplementary Figure S4. Neural efficiency in the left medial PFC for each trial, where the individual points are the average for each trial in each condition, and the solid lines indicate the model's prediction.

Right Medial Prefrontal Cortex

The average total hemoglobin concentration in the right medial prefrontal cortex was approaching difference from zero in the Standard condition (β=0.74, SE=0.39, p=0.06). The Vibrotactile condition was not significantly different from the Standard condition (β=-0.27, SE=0.46, p=0.55). Similarly, the Haptic Shared Control condition was not significantly different from the Standard condition (β=-0.57, SE=0.46, p=0.22). Experience with the task approached significance in reducing cognitive load (approaching significant change in total hemoglobin concentration: β=-0.09, SE=0.05, p=0.06). See Fig. S5 for a visualization of these results.

Supplementary Figure S5. The change in average total hemoglobin concentration in the right medial PFC for each trial, where the individual points are the average for each trial in each condition, and the solid lines indicate the model's prediction.

The neural efficiency in the right medial prefrontal cortex was significantly less than zero in the Standard condition (β=-0.84, SE=0.28, p=0.005). The Vibrotactile condition was not significantly different from the Standard condition (β=0.29, SE=0.35, p=0.41). However, the Haptic Shared Control condition significantly improved the neural efficiency compared to the Standard condition (β=0.72, SE=0.35, p=0.048). Experience with the task improved the neural efficiency: β=0.12, SE=0.03, p<0.001). See Fig. S6 for a visualization of these results.

Supplementary Figure S6. Neural efficiency in the right medial PFC for each trial, where the individual points are the average for each trial in each condition, and the solid lines indicate the model's prediction.
